# Practice development perspective of RTT contouring in online adaptive radiotherapy for prostate cancer: A single-centre cost-consequence analysis

**DOI:** 10.1016/j.tipsro.2026.100391

**Published:** 2026-03-12

**Authors:** Bethany Williams, Emma Oi Ching Xue, Alison Tree, Helen McNair, Kyriaki Giorgakoudi

**Affiliations:** a Department of Population Health and Policy, School of Health and Medical Sciences, City St George’s, University of London, UK; b The Royal Marsden NHS Foundation Trust, UK; c The Institute of Cancer Research, UK; d NIHR Biomedical Research Centre at The Royal Marsden NHS Foundation Trust and The Institute of Cancer Research, UK

**Keywords:** RTT contouring, Online adaptive radiotherapy, Resources, Cost saving, RTT role expansion, RTT-led practice

## Abstract

Technological advances in online adaptive radiotherapy (oART) are set to revolutionise the treatment of prostate cancer (PCa). Yet, the need for a multi-disciplinary team oversight at every fraction remains a significant resource barrier to wider implementation. This practice-development paper explores the cost-consequence analysis and the operational implications of delegating online contouring responsibilities to therapeutic radiographers (RTTs) within an established MRI-guided online adaptive radiotherapy (oART) PCa service. Using a discrete-event simulation model informed by single-centre workflow data, the implications of RTT- and radiation oncologist (RO)-contoured workflows are discussed in terms of personnel costs, RO time, and patient throughput. With RTT online contouring generating substantial cost savings, demonstrating how reallocation of tasks can improve service efficiency and support sustainable oART. Highlighting the importance of workforce development and economic evidence to help to inform policy decisions, with the aim to broaden access to adaptive radiotherapy worldwide.

## Highlights

• RTT online contouring provides cost savings in online adaptive prostate radiotherapy.
• Increasing RTT scope of practice results in time saving efficiencies for radiation oncologists.
• RTT availability increases patient throughput compared to radiation oncologists.
• RTT role expansion eases pressure on healthcare resources in online adaptive radiotherapy.

## Introduction

Radical radiotherapy remains the mainstay of curative treatment for over two-thirds of male patients diagnosed with prostate cancer (PCa) in England and Wales [1]. Advances in online adaptive radiotherapy (oART) aim to improve treatment precision and patient health outcomes. Magnetic resonance image-guided radiotherapy (MRIgRT) combines MRI and daily online adaptive planning to account for inter- and intra-fractional anatomical variation [2]. In PCa oART, the increased accuracy has been demonstrated to enable margin reduction and ultra-hypofractionation with reported reduction in genitourinary adverse events [3], [4].

Despite clinical advantages, oART remains resource-intensive compared to conventional radiotherapy, requiring the involvement of radiation oncologists (RO), therapeutic radiographers (RTT), and medical physicists at each treatment. Specifically, this pathway relies on ROs to conduct online contouring of target volumes and organs at risk (OAR), as well as plan approval [5]. This poses a significant barrier to broader implementation amidst the global challenge of increasing cancer incidence, rising waiting times and constrained resources [1].

However, emerging practice development initiatives present opportunities to redefine professional roles. Among these include the delegation of online contouring to RTTs and plan approval to a delegated operator. While previous studies have explored the feasibility and accuracy of RTT contouring [5], [6], [7], [8], [9], [10], a gap remains in addressing the wider practice development implications, specifically the health economic impact of adopting this innovative workflow.

As a centre with a well-established RTT-contoured service for PCa MRIgRT on the Elekta Unity MR-Linac (MRL) (Elekta AB, Stockholm, Sweden), this practice development perspective presents a cost-consequence analysis (CCA) and explores the operational implications of RTT online contouring. With intention to inform service development, and support evidence-based policy decisions around RTT role expansion, this work supports broader implementation and increased patient access to oART.

### Practice development: RTT role expansion

Successfully shifting from traditional multi-disciplinary attendance at every adaptive fraction requires substantial RTT credentialing and a robust training framework. Specifically, RTTs must be upskilled to undertake roles traditionally held by RO and medical physicists, including contouring, treatment planning and plan approval. Current literature and community collaboration provides guidance on oART advanced skills credentialling [10], [11], [12] and reinforces that RTTs can be successfully trained in these activities, including leadership of all tasks in RTT-led oART [10], [12], [13], [14].

Numerous international centres have implemented RTT online contouring for MRIgRT of PCa, as well as other treatment indications [5], [6], [7], [8], [9], [10]. Validation of RTT target volume and OAR contouring is frequently demonstrated through volumetric comparison of RTT to RO contours [5], [6], [7], [8], [9], [10]. Additional studies demonstrate clinically acceptable dosimetric deviation in oART plans [5], [6], [7]. Collectively, this evidence proves that RTTs can be competently trained to extend their scope to contouring.

We implemented RTT online contouring for long-course oART of PCa (20-fractions) in 2021, with the well-established service extended to hypo-fractionated stereotactic oART (5-fractions) in 2022. Plan approval is delegated to medical physicists. The implementation has released ROs from online workflows, requiring their presence at fraction 1 only [5]. RTT role reallocation reduces oART resource intensity towards a more flexible, sustainable workforce model. Yet, barriers remain. As an emerging technology a global oART skills deficit exists for RTT-led contouring and treatment planning as well as resistant attitudes towards role reallocation and restrictions on practice by governing bodies or varying international jurisdiction [11]. This highlights the need for economic evaluation to inform policy, support the upskilling of RTTs and to advocate sustainable workforce models in oART.

Broader healthcare literature demonstrates the economic value of extending allied health professional scope. Examples include the economic benefit of diagnostic radiographers taking on MRI reporting from radiologists [15]. Likewise, the cost and consequential impact of non-medical prescribing roles, where three of nine studies in scoping review showed pharmacist prescribing was superior in all patient outcomes and cost saving at a large scale. The remaining studies reported similar results in most outcomes across other non-medical prescribers [16]. These studies support the expansion of allied health professional scope and emphasise the importance of further research into RTT task shifting in oART.

### Model development reporting cost-consequence in RTT-contoured workflows

Economic evaluations in radiotherapy traditionally scope their analyses to compare treatment modalities and fractionation regimens. To our knowledge, practice development implications of changes in operational workflows that involve task shifting within radiotherapy teams have not been previously addressed in a CCA. Several studies have instead, compared the costs between MRI-guided versus computed tomography-guided adaptive radiotherapy (CTgART) [17], [18], [19]. One economic evaluation reported MRIgRT to be cost-effective for localised PCa through minor toxicity reductions [17]. Previous studies could be strengthened by incorporating a CCA of task shifting into their economic analyses, as this may influence the overall cost-effectiveness of MRIgRT.

Two of these studies used a time-driven activity-based costing (TDABC) methodology [18], [19]. While TDABC typically calculates costs using average times and resource use, alternative discrete event simulation (DES) models simulate the stochastic nature of real-world processes [20], [21], [22], [23]. Thus, enabling representation of inherent variability in clinical workflows more accurately and has been widely used in the field of radiotherapy process analysis [24], [25]. This extensive application and the model’s ability to capture complex, real-life scenarios underpin preference for a patient-level time-to-event simulation model over methods such as TDABC and traditional modelling health economic approaches.

A DES model was constructed using Simul8 2022 (Simul8 Corporation, Boston, MA, USA) based on workflow timing data from this single-centre setting (Supplementary A, Fig. A1). Workflow stages from patient arrival to departure, included patient positioning, image acquisition, contouring, plan optimisation, image verification and treatment delivery. The possibility of accounting for intrafraction motion was also included. Models for the RTT contouring and RO contouring were created (Supplementary B Figs. B1 and B2). The RTT contouring model (Supplementary B Fig. B1) includes a RO contouring pathway, to represent ROs contouring the first fraction of treatment. Resources incorporated in the models include personnel involved in the workflow, including ROs, RTTs and medical physicists (Supplementary C Table C1). To capture the data’s inherent variability, the activities in the models were populated with probability distributions derived from observed data. RStudio (version 4.3.1) was used to determine the distribution functions that best fit the timing data for each activity in the models (Supplementary D).

Personnel costs were sourced from the Personal Social Services Research Unit (PSSRU), in accordance with the National Institute for Health and Care Excellence (NICE) guidelines from a healthcare payer’s perspective [26], [27]. Costs were estimated in 2022 British pounds sterling (£). To reflect the local setting, these costs were inflated by adding a location-based weighting [28] (Supplementary E Table E1). The hourly rates for each staff shown in Table 1 were converted into cost per second and included in the Simul8 model.

**Table 1:** Staff unit costs.

Staff	Unit	Cost (£)	Source
RO	Cost/working hour	148	[26], [28] Details in Supplementary E
Medical physicist	Cost/working hour	67	[26], [28] Details in Supplementary E
RTT	Cost/working hour	67	[26], [28] Details in Supplementary E

This analysis focusses on personnel costs, while excluding costs associated with physics, capital outlay of the machine, servicing, and running costs. These elements, which represent a large share of overall cost of the MRL, are expected to be comparable across both workflows. By excluding these costs, the reallocation of the task of contouring is evaluated only.

The models were run for a cohort size of 58 based on the number of patients referred for radiotherapy to the prostate and seminal vesicles alone and eligible for MRIgRT at this centre in the 2022–23 financial year. The model was validated as per Supplementary G.

### Cost savings: A single-centre setting

In application of this example model to this single-centre setting, the RTT contouring workflow is estimated to lead to a per-patient cost saving of £1,867 for the 20-fraction regimen, which equates to a cumulative cohort cost reduction of £108,311 (Table 2). If patients were treated using a 5-fraction regimen instead, the per-patient cost saving would be £421, corresponding to a total cohort saving of £24,440. The higher cost per-fraction for 5-fraction regimen is due to inherently longer workflow duration than 20-fraction indicated by workflow timing data. The increased workflow duration is not only due to marginally increased contouring time, but longer plan optimisation and treatment delivery resulting in prolonged workflow and RO presence.

**Table 2:** Cost analysis of comparing RO contouring and RTT contouring at single-centre.

	20-Fraction Regimen			5-Fraction Regimen		
	RO Contouring	RTT Contouring	Difference between RO contouring and RTT contouring	RO Contouring	RTT Contouring	Difference between RO contouring and RTT contouring
Costs (£) (95% confidence intervals)						
Cost/fraction	244.77	151.40	93.37	293.74	209.47	84.28
Cost/patient	4,895.48	3,028.06	1,867.42	1468.71	1,047.33	421.38
Total cohort cost	283,937.84 (282,806.71–285,068.97)	175,627.27 (173,653.06–177,601.47)	108,310.57	85,185.39 (82,637.83–87,732.96)	60,745.20 (59,957.72–61,532.68)	24,440.19

### Operational consequences: A single-centre setting

Beyond cost, delegation of online contouring to RTTs yields RO time efficiencies. From the perspective of the duration from patient arrival to the beginning of treatment delivery where the RO is present (‘RO attendance’), the model estimates a total cohort saving of 685 h of RO time can be saved for 20-fraction treatments and 167 h for the 5-fraction treatments (Table 3).

**Table 3:** Consequence analysis of comparing RO contouring and RTT contouring at single-centre.

	20-Fraction Regimen			5-Fraction Regimen		
	RO Contouring	RTT Contouring	Difference between RO contouring and RTT contouring	RO Contouring	RTT Contouring	Difference between RO contouring and RTT contouring
Consequences						
Total ‘RO attendance’ time in minutes [hours]	43,193.2 [719.9]	2,117.1 [35.3]	41,076.1 [684.6]	12,542.4 [209.0]	2,506.1 [41.8]	10,036.3 [167.3]
Total ‘RO active’ time in minutes [hours]	29,032.6 [483.9]	1,437.4 [24.0]	27,595.2 [459.9]	9,020.8 [150.4]	1,824.9 [30.4]	7,195.9 [119.9]

While ROs are involved in online contouring and plan approval only, attending in person, they are prevented from productive engagement in other tasks. However technological advances have enabled remote access to online workflows, whereby ROs can perform oART responsibilities from their office, clinic or home. When considering the period in which ROs are actively engaged in the workflow (‘RO active’), saving remains substantial at 460 h and 120 h for the 20-fraction and 5-fraction regimens, respectively (Table 3).

Appointment scheduling and the machine’s maximum capacity for clinical use (clinical service hours) were based on clinical practice, informed by workflow timing data, with appointments set at one-hour intervals. This schedule results in a maximum weekly capacity of 40 h on the machine for the RTT contouring model. The RO contouring model was run to accommodate 16 appointments per week on the MRL, reflecting the declared availability of urology specialist ROs or clinical fellows who collectively cover all prostate treatments. These capacities correspond to 2,080 and 832 annual clinical service hours for the RTT and RO models, respectively (Supplementary F), reflecting a potential 150% increase in annual machine capacity. It is acknowledged that the availability of urology ROs is specific to this single-centre and a primary factor in resultant capacity, which may vary between centres yet exemplifies potential impact of RTT contouring on patient throughput.

In our DES model, we implemented an 8-hour daily schedule for the staff in the RTT online contouring workflow, spanning the entire week. Similarly, for the RO online contouring model, staff were assigned 8-hour workdays but limited to two days per week. Reflecting an increase in potential maximum annual patient throughput; specifically, 63 more patients for 20-fractions and 250 more patients for 5-fractions, where PCa is assumed the only treated indication. This approach is a simplification adopted for modelling, we acknowledge that MRL treatments extend beyond just PCa but this estimation provides an indication of potential increase in patient throughput with RTT contouring if we assume similar efficiencies across all MRL treatment indications. Alternatively, by utilising clinical service hours to estimate capacity of the MRL and accounting that prostate treatments represented 53.2% of its utilisation in the financial year 2022–23, the increase in annual patient throughput could be estimated as: 33 patients for 20-fractions and 132 patients for 5-fractions.

### Single-centre experience and implications of practice development

This application of a DES model to this single-centre demonstrates significant benefits of the practice development of delegating online contouring responsibilities to RTTs in MRIgRT for PCa. This shift results in cost savings, sparing oART service expenses or allowing the NHS to reallocate funds to other areas. Additionally, this leads to reductions in RO time, compared to a RO-contoured workflow. This valuable time can be redirected to key RO responsibilities, including patient consultations, follow ups in clinics, multidisciplinary team meetings, and research. OART treatment duration can be significantly longer than conventional radiotherapy; yet current RO job plans rarely account for the shift in workload. Given the increasing validation of RTT online contouring, the practice of requiring an RO at the first treatment may be phased out [6], further sparing cost and time resources. Alternatively, some centres have invested in employment of ROs specifically to adaptive platforms or scheduling an ‘RO of the day’ whereby one RO is available for all online treatments scheduled in one working day. However, this practice development perspective demonstrates how personnel costs could be otherwise saved.

Furthermore, this centre reports increased patient throughput following implementation of RTT contouring. With RTT-led contouring, referrals are no longer constrained by RO schedules, with capacity limited only by clinical service hours and demand. These capacities correspond to an increase in potential patient throughput based on annual clinical service hours and RTT versus RO availability. Notably, simplification adopted for the purposes of modelling machine capacity assumes PCa is the only treatment indication referred, while this is clearly not the case, results indicate the potential increase in machine capacity if RTT contouring was extended to other treatment sites.

Other studies have reported that with experience and more frequent software exposure RTTs can become more efficient at contouring than ROs [7], [14]. This, alongside potential for increased patient throughput improves patient access to care, potentially reducing waiting times. The increased availability of RTTs compared to ROs improves appointment flexibility, a benefit for patient experience and in turn increases capacity for alternative treatment indications where a RO is required.

### Implications of practice development for clinical practice and healthcare policy

RTT online contouring has notable implications for clinical practice and healthcare policy, especially considering the universal budget constraints faced by healthcare systems. Substantial cost savings, reduction of RO time, and potential for increased patient throughput, backs re-evaluation of traditional roles towards RTT online contouring and RTT-led practice.

These insights underscore need for further research into training, advanced practice and role flexibility across healthcare settings, especially in the context of chronic staff shortages and the imperative to optimise resource allocation. The RTT profession is at risk globally, expanding RTT scope may facilitate staff recruitment and retention [11]. This is demonstrated by an existing strong demand for training and education in oART, with one UK based study reporting MRI contouring as one of the highest priority needs [29]. These findings are crucial in guiding health policy and practice in undergraduate, postgraduate and clinical settings.

Further research may also influence reconsideration of current oART reimbursement models. The existing tariff structures may not effectively reflect the cost dynamics of increasingly popular hypo-fractionated treatments [30]. This misalignment could disincentivise providers from adopting such treatments. The issue stems from current tariffs potentially not always offsetting the higher upfront costs, thus not consistently resulting in cost savings for providers.

While the CCA presented provides valuable insights, the findings should be considered within the context its specific hospital setting. A limitation in our analysis is that the number of observations is small for some parameters of interest. Estimations on implications on machine capacity do not consider machine servicing, upgrades or breakdowns. In addition, the scope of our analysis did not account for costs associated with training; however, these are acknowledged as essential factors and should be considered in future economic evaluations.

It is crucial to consider future economic evaluations of treatment sites other than PCa, which will help determine the broader implications of online RTT contouring in MRIgRT, as well as alternative platforms, such as CTgART. Alternatively, other RTT-led workflows, including in different contexts, in local, national and international settings. By exploring these comparisons, the findings have the potential to contribute to the evidence base supporting the wider adoption of oART and optimisation of treatment approaches through sustainable staffing models, helping to challenge resistance towards RTT role expansion and shape healthcare policies on a national and international scale.

## Conclusion

This practice development paper contributes a health economic perspective to the growing body of literature focusing on optimising oART through RTT-led practice. It highlights the economic and efficiency benefits of transitioning from RO to RTT online contouring, showing role reallocation can streamline oART workflows, evidenced by cost savings and time efficiencies. It may also increase patient throughput due to expanded capacity, mitigating bottlenecks caused by RO scheduling constraints. These findings are crucial in guiding health policy and practice, with potential impact on lack of investment and resources in training, and RTT retention. Ultimately, this work facilitates more sustainable staffing in oART, with potential to increase patient accessibility to advanced treatments worldwide.

## Author contributions

Bethany Williams: Conceptualization, Data curation, Methodology, Writing – original draft, Writing – review and editing. Emma Xue: Conceptualization, Data curation, Formal analysis, Investigation, Methodology, Writing – original draft, Writing – review and editing. Alison Tree: Writing – review and editing. Kyriaki Giorgakoudi: Conceptualization, Methodology, Writing – review and editing. Helen McNair: Conceptualization, Funding acquisition, Methodology, Writing – review and editing.

## Funding

This publication was funded by the National Institute for Health Research (ICA-SCL-2018–04-ST2-002).

## Declaration of competing interest

The authors declare the following financial interests/personal relationships which may be considered as potential competing interests: This project is independent research funded by the National Institute for Health Research and Health Education England (HEE/NIHR Senior Clinical Lectureship, Helen McNair, ICA-SCL-2018-04-ST2-002).

A. Tree is supported by a Cancer Research UK Radiation Research Centre of Excellence at The Institute of Cancer Research and The Royal Marsden NHS Foundation Trust (grant refs: A28724 and RRCOER-Jun24/100006) and a Cancer Research UK Programme Grant (ref: C33589/A28284).

A. Tree, H. A. McNair and B. Williams acknowledge NHS funding to the NIHR Biomedical Research Centre at The Royal Marsden and The Institute of Cancer Research.

H. A. McNair and B. Williams are funded by The Royal Marsden Cancer Charity.

KG is supported by the National Institute for Health Research (NIHR) Biomedical Research Centre at The Royal Marsden NHS Foundation Trust and the Institute of Cancer Research, London.

The views expressed in this publication are those of the author(s) and not necessarily those of the NHS, the National Institute for Health Research or the Department of Health and Social Care.

The Royal Marsden Hospital and The Institute of Cancer Research are members of the Elekta MR-Linac Consortium, which aims to coordinate international collaborative research relating to the Elekta Unity (MR- Linac). Elekta (Elekta AB, Stockholm, Sweden) and Philips (Philips, Best, the Netherlands) are commercial members of the MR-Linac Consortium. Elekta financially supports consortium member institutions with research funding, education and travel costs for consortium meetings.
