# Supplementary material for: Practice development perspective of RTT contouring in online adaptive radiotherapy for prostate cancer: A single-centre cost-consequence analysis
Source: Tech Innov Patient Support Radiat Oncol. 2026 Mar 12;38:100391. doi: 10.1016/j.tipsro.2026.100391 (PMC13010981; doi:10.1016/j.tipsro.2026.100391)
Supplement: Supplementary Data 1 [file mmc1.docx]

Supplementary Material

# Supplementary A – Data collection and data collection sheet


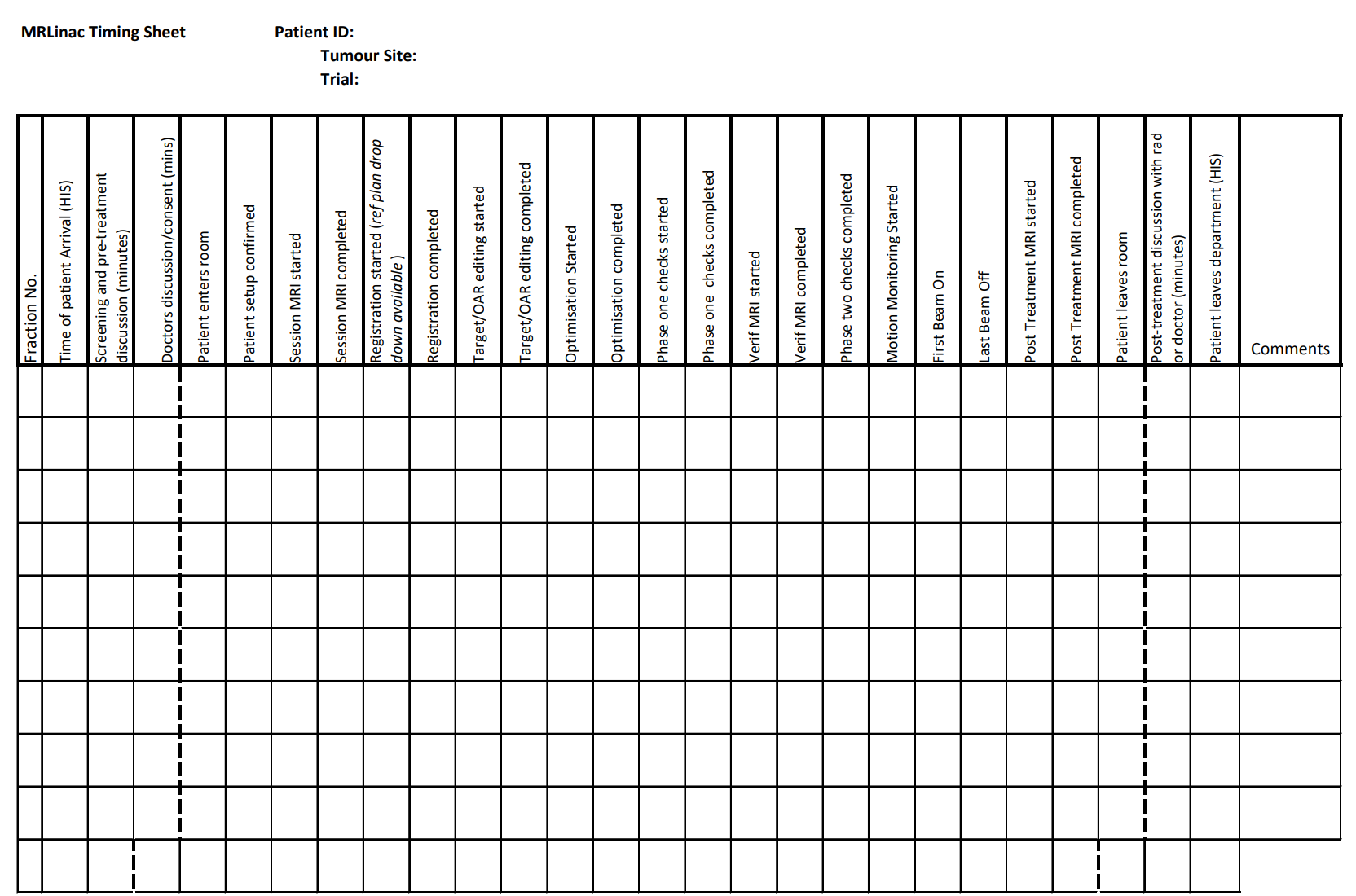
Workflow analysis to inform model development was based on a dataset comprised of timing data from 370 fractions from 31 patients. From the 20-fraction regimen, data were available from 296 fractions (136 contoured by ROs, 160 by RTTs) across 15 patients treated between. For the 5-fraction regimen, we had observations from 74 fractions (59 contoured by ROs, 15 by RTTs) across 16 patients treated between.

Supplementary A Figure A1. Timing sheet

#
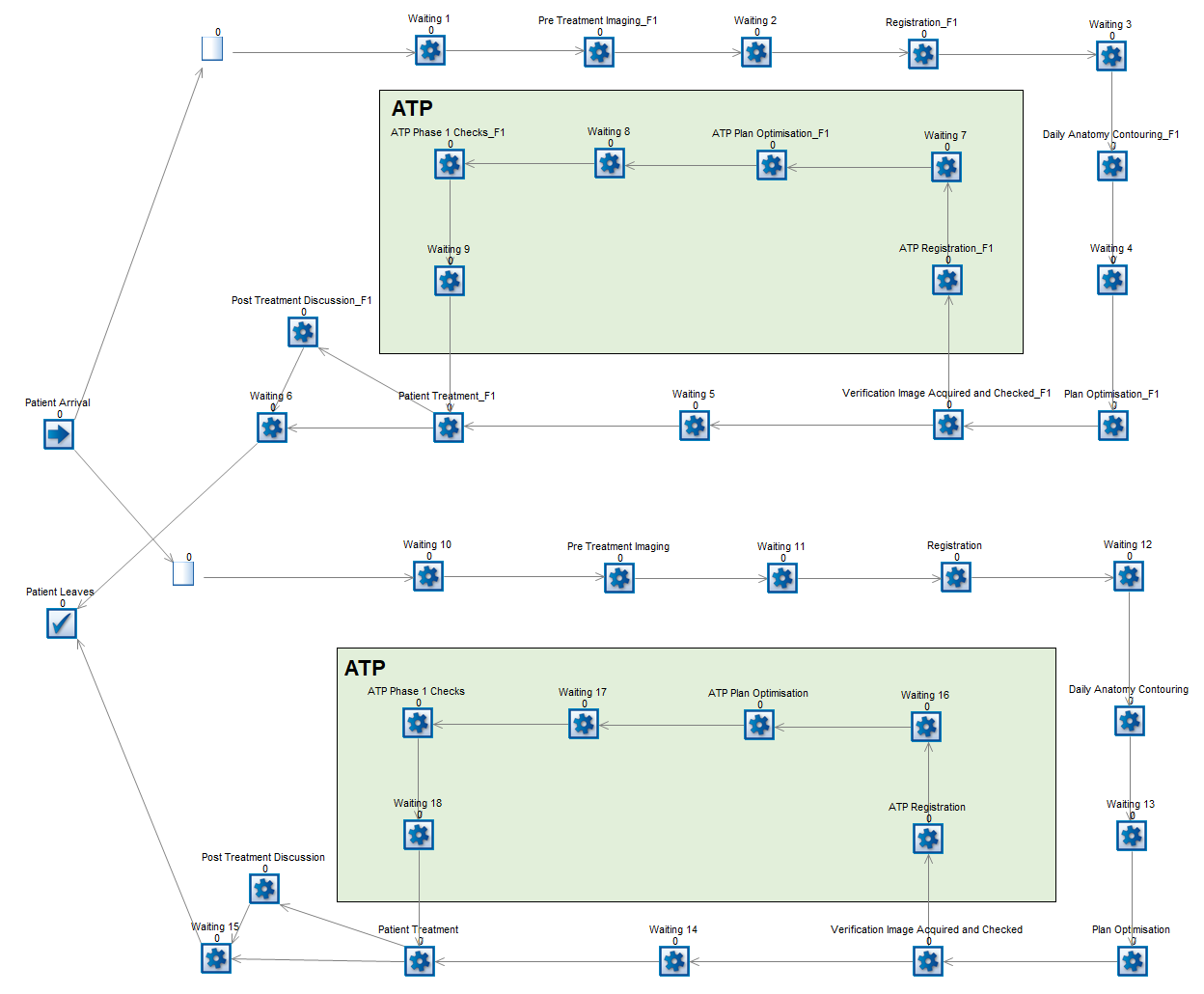
Supplementary B – Simul8 models

Supplementary B, Figure B1. Simul8 DES RTT contouring model

Supplementary B, Figure B2. Simul8 DES radiation oncologist contouring model
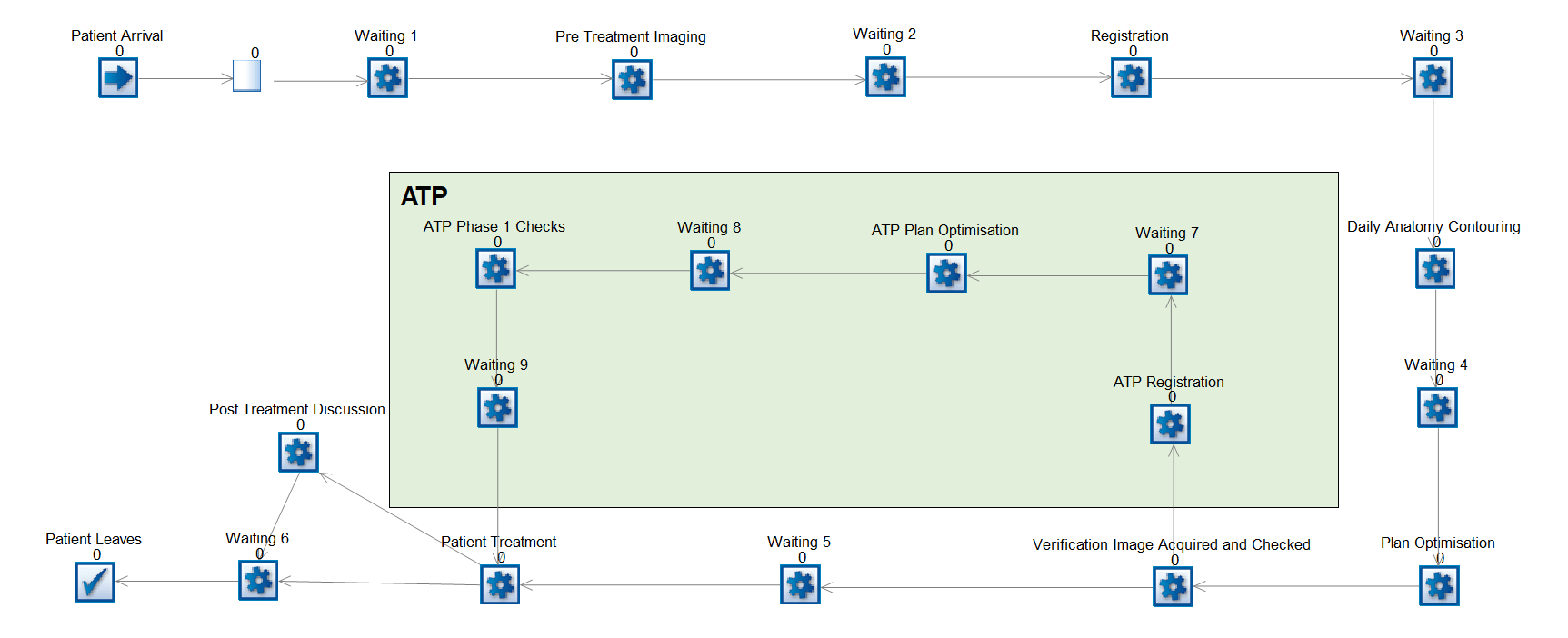
**.**

**Supplementary C – Staff presence in model**

Supplementary C Table C1. Staff presence in the model

| Activity block | RO | Medical physicist | RTT |
| --- | --- | --- | --- |
| **RO contouring model** | | | |
| Waiting 1* | 1 | 1 | 2 |
| Pre Treatment Imaging | 1 | 1 | 2 |
| Waiting 2 | 1 | 1 | 2 |
| Registration | 1 | 1 | 2 |
| Waiting 3 | 1 | 1 | 2 |
| Daily Anatomy Contouring | 1 | 1 | 2 |
| Waiting 4 | 1 | 1 | 2 |
| Plan Optimisation | 1 | 1 | 2 |
| Verification Image Acquired and Checked | 1 | 1 | 2 |
| Waiting 5 | 1 | 1 | 2 |
| Patient Treatment | 0 | 1 | 2 |
| Post Treatment Discussion | 0 | 0 | 1 |
| Waiting 6 | 0 | 0 | 2 |
| ATP Registration | 1 | 1 | 2 |
| Waiting 7 | 1 | 1 | 2 |
| ATP Plan Optimisation | 1 | 1 | 2 |
| Waiting 8 | 1 | 1 | 2 |
| ATP Phase 1 Checks | 1 | 1 | 2 |
| Waiting 9 | 1 | 1 | 2 |
| **RTT contouring model** | | | |
| Waiting 1 | 1 | 1 | 2 |
| Pre-treatment imaging_F1 | 1 | 1 | 2 |
| Waiting 2 | 1 | 1 | 2 |
| Registration_F1 | 1 | 1 | 2 |
| Waiting 3 | 1 | 1 | 2 |
| Daily Anatomy Contouring_F1 | 1 | 1 | 2 |
| Waiting 4 | 1 | 1 | 2 |
| Plan Optimisation_F1 | 1 | 1 | 2 |
| Verification Image Acquired and Checked_F1 | 1 | 1 | 2 |
| Waiting 5 | 1 | 1 | 2 |
| Patient Treatment_F1 | 0 | 1 | 2 |
| Post Treatment Discussion_F1 | 0 | 0 | 1 |
| Waiting 6 | 0 | 0 | 2 |
| ATP Registration_F1 | 1 | 1 | 2 |
| Waiting 7 | 1 | 1 | 2 |
| ATP Plan Optimisation_F1 | 1 | 1 | 2 |
| Waiting 8 | 1 | 1 | 2 |
| ATP Phase 1 Checks_F1 | 1 | 1 | 2 |
| Waiting 9 | 1 | 1 | 2 |
| Waiting 10 | 0 | 1 | 2 |
| Pre Treatment Imaging | 0 | 1 | 2 |
| Waiting 11 | 0 | 1 | 2 |
| Registration | 0 | 1 | 2 |
| Waiting 12 | 0 | 1 | 2 |
| Daily Anatomy Contouring | 0 | 1 | 2 |
| Waiting 13 | 0 | 1 | 2 |
| Plan Optimisation | 0 | 1 | 2 |
| Verification Image Acquired and Checked | 0 | 1 | 2 |
| Waiting 14 | 0 | 1 | 2 |
| Patient Treatment | 0 | 1 | 2 |
| Post Treatment Discussion | 0 | 0 | 1 |
| Waiting 15 | 0 | 0 | 2 |
| ATP Registration | 0 | 1 | 2 |
| Waiting 16 | 0 | 1 | 2 |
| ATP Plan Optimisation | 0 | 1 | 2 |
| Waiting 17 | 0 | 1 | 2 |
| ATP Phase 1 Checks | 0 | 1 | 2 |
| Waiting 18 | 0 | 1 | 2 |

*’Waiting’ blocks are incorporated here to represent the time intervals between activities. Patient set-up time is incorporated within ‘waiting 1’, which also includes the time from patient set-up confirmation to the start of pre-treatment imaging. The model does not explicitly include patient set-up as a separate activity because the provided data only included the patient ready/patient set-up confirmed time without the start time for the set-up process.

# Section D – Probability distributions and parameter estimates

To capture the data’s inherent variability, the activities in the models were populated with probability distributions derived from observed data. RStudio (version 4.3.1) was used to determine the distribution functions that best fit the timing data for each activity in the models. Supplementary D Table D1 shows the distribution for each activity and the corresponding parameter values used in the distribution function in Simul8. These were derived through a combination of complementary methods. Histograms were plotted initially to visualise the spread of data. The R package ‘fitur’ enabled further data visualisation through probability-probability (P-P) plots and quantile-quantile (Q-Q) plots, and to check goodness of fit through the Anderson-Darling test. Goodness of fit was further assessed through comparing Akaike Information Criterion (AIC) and Bayesian Information Criterion (BIC) values. These collective methods of data visualisation and the Anderson-Darling test were used initially to identify the best fitting distributions for each activity, then AIC and BIC served as confirmatory measures. Subsequently, the selected distributions were specified and included into the Simul8 model. Due to the limited number of observations in the data for the post-treatment discussion parameter, a probability distribution was not fitted. Instead, a fixed (mean) value was calculated and used.

Data for RO contouring and RTT contouring were analysed separately to derive parameter estimates for the different models. Given the limited availability of data for the 5-fraction treatments contoured by RTTs, data for 20-fraction and 5 fraction treatments were combined for most activities, under the assumption that their timings would be comparable. However, for certain activities where distinctions are anticipated, the 20-fraction and 5-fraction treatments data were analysed separately. These were for daily anatomy contouring (acknowledging additional urethra OAR for 5-fraction), plan optimisation, patient treatment and plan optimisation in the ATP pathway. Data for the ATP pathway in both 20-fraction and 5-fraction treatments were collectively analysed, regardless of whether the fraction was contoured by the RO or RTTs. Since this process is always handled by RTTs, no differences are expected, so merging the data provides a more thorough analysis.

Supplementary Table D1. Model inputs of probability distributions and parameter estimates

| Parameter | Distribution | Parameter estimates required for model |
| --- | --- | --- |
| **RO 5# and RO 20#** | | |
| Patient consultation | Lognormal | Mean=196.00, SD=87.93 |
| Waiting 1 (wait between patient consultation and pre-treatment imaging) | Exponential | Mean=272.27 |
| Pre-treatment imaging | Lognormal | Mean=173.67, SD=50.73 |
| Waiting 2 (wait between pre-treatment imaging and registration) | Gamma | Alpha=6.42, beta=15.74 |
| Registration | Weibull | Alpha=2.81, beta=120.86, minimum=30 |
| Waiting 3 (wait between registration and daily anatomy contouring) | Gamma | Alpha=2.57, beta=19.90 |
| Waiting 4 (wait between daily anatomy contouring and plan optimisation) | Normal | Mean=162.54, SD=61.66 |
| Verification image acquired and checked | Lognormal | Mean=264.87, SD=85.75 |
| Waiting 5 (wait between verification image acquired and checked and patient treatment) | Lognormal | Mean=57.04, SD=79.70 |
| Post-treatment discussion | Fixed value | Mean=90 |
| Waiting 6 (wait between patient treatment/post-treatment discussion and patient leaves) | Lognormal | Mean=206.01, SD=123.205 |
| **RO 5#** | | |
| Daily anatomy contouring | Gamma | Alpha=11.08, beta=69.94 |
| Plan optimisation | Normal | Mean=342.66, SD=63.37 |
| Patient treatment | Normal | Mean=616.64, SD=91.35 |
| **RO 20#** | | |
| Daily anatomy contouring | Lognormal | Mean=729.72, SD=239.20 |
| Plan optimisation | Gamma | Alpha=17.75, beta=11.88 |
| Patient treatment | Lognormal | Mean=366.77, SD=38.55 |
| **RTT 5# and RTT 20#** | | |
| Patient consultation | Lognormal | Mean=199.57, SD=101.76 |
| Waiting 1 (wait between patient consultation and pre-treatment imaging) | Normal | Mean=227.37, SD=109.46 |
| Pre-treatment imaging | Normal | Mean=196.30, SD=63.17 |
| Waiting 2 (wait between pre-treatment imaging and registration) | Normal | Mean=93.98, SD=37.24 |
| Registration | Normal | Mean=107.51, SD=36.89 |
| Waiting 3 (wait between registration and daily anatomy contouring) | Exponential | Mean=63.86 |
| Waiting 4 (wait between daily anatomy contouring and plan optimisation) | Lognormal | Mean=200.00, SD=93.31 |
| Verification image acquired and checked | Lognormal | Mean=270.63, SD=82.88 |
| Waiting 5 (wait between verification image acquired and checked and patient treatment) | Exponential | Mean=63.09 |
| Post-treatment discussion | Fixed value | Mean=120 |
| Waiting 6 (wait between patient treatment/post-treatment discussion and patient leaves) | Lognormal | Mean=186.83, SD=103.68 |
| **RTT 5#** | | |
| Daily anatomy contouring | Lognormal | Mean=659.97, SD=291.08 |
| Plan optimisation | Weibull | Alpha=5.90, beta=320.88, minimum=154.00 |
| Patient treatment | Lognormal | Mean=679.06, SD=123.77 |
| **RTT 20#** | | |
| Daily anatomy contouring | Gamma | Alpha=9.05, beta=82.34 |
| Plan optimisation | Gamma | Alpha=16.87, beta=12.43 |
| Patient treatment | Lognormal | Mean=369.28, SD=54.01 |
| RO 5# and RTT 5# |  |  |
| ATP plan optimisation | Lognormal | Mean=165.12, SD=35.40 |
| **RO 20# and RTT 20#** | | |
| ATP plan optimisation | Lognormal | Mean=69.89, SD=38.35 |
| **RO 5#, RO 20#, RTT 5# and RTT 20#** | | |
| ATP registration | Gamma | Alpha=4.13, beta=22.46 |
| Waiting 7 (wait between ATP registration and ATP plan optimisation) | Lognormal | Mean=67.10, SD=53.01 |
| Waiting 8 (wait between ATP plan optimisation and ATP phase 1 checks) | Lognormal | Mean=64.51, SD=39.77 |
| ATP phase 1 checks | Gamma | Alpha=2.49, beta=16.85 |
| Waiting 9 (wait between ATP phase 1 checks and patient treatment) | Exponential | Mean=21.36 |

# Supplementary E – Calculation of personnel unit costs

Supplementary E Table E1. Breaking down calculation of staff unit costs with a London weighting

|  | RO | Medical physicist | RTT | Source/Explanation |
| --- | --- | --- | --- | --- |
| Total costs per year (£) | 300,173 | 102,728 | 102,728 | [25] Using Consultant: medical for RO costs.  Using band 7 for medical physicist and RTT costs. |
| London weighting (£) | 5,436 | 5,436 | 5,436 | [27] Using the Outer London Weighting of 15% of basic salary, with a maximum payment of £5,436 |
| Total cost + London weighting (£) | 305,609 | 108,164 | 108,164 |  |
| Working hours per year | 2,070 | 1,613 | 1,613 | [25] |
| Cost per working hour (£) (to nearest integer) | 148 | 67 | 67 | (Total cost + London weighting)/working hours per year |

# Supplementary F – Calculation of patient throughput based on clinical service hours

Clinical service hours is defined as the hours the machine is available for clinical treatments. In our main analysis, we have outlined that:

- The RTT contouring model utilises the MRL for 40 hours per week.
- The RO contouring model utilises the MRL for 16 hours per week.

RTT contouring model:

Annual clinical service hours:

$$40 hours per week*52 weeks=2,080 hours per annum$$

RO contouring model:

Annual clinical service hours:

$$16 hours per week*52 weeks=832 hours per annum$$

# Section G – Model validation

The structure of the model was periodically reviewed by RTTs involved in the study, whose feedback was incorporated to ensure the model reflected an accurate simulation to the real-life clinical process. The model parameters were validated through comparing the simulation output mean and 95% confidence intervals with the observed mean values. The 95% confidence intervals were determined by running the models for a set number of replications recommended by Simul8’s trial calculator function, which suggested four replications.

To validate our model, the estimated mean timings were compared with their corresponding 95% confidence intervals from the simulation for both RO contouring and RTT contouring against observed means (Supplementary G Table G1). Minor disparities existed between the simulated and observed means, with deviations ranging from a minimum of 14 seconds to a maximum of 186 seconds outside the 95% confidence intervals. Despite these discrepancies, the proximity between the model means and observed means suggests a robust output from the model.

Supplementary G Table G1. Comparison between simulation output mean and observed data mean (time in minutes)

|  | 20-Fraction Regimen | | 5-Fraction Regimen | |
| --- | --- | --- | --- | --- |
|  | RO Contouring | RTT Contouring | RO Contouring | RTT Contouring |
| Model mean (95% CI) | 46.83 (46.64-47.03) | 44.03 (43.86-44.21) | 56.90 (56.43-57.37) | 57.28 (56.85-57.72) |
| Observed mean | 46.32 | 46.20 | 60.47 | 56.62 |
| *CI = Confidence interval | | | | |
